# Hypotonic male infant and MCT8 deficiency - a diagnosis to think about

**DOI:** 10.1186/1471-2431-14-252

**Published:** 2014-10-04

**Authors:** Filipa Rodrigues, Joana Grenha, Carlos Ortez, Andrés Nascimento, Beatriz Morte, Monica M-Belinchón, Judith Armstrong, Jaume Colomer

**Affiliations:** Neuromuscular Unit, Neurology Department, Fundación Sant Joan de Déu, Hospital Materno-Infantil Sant Joan de Déu, Passeig Sant Joan de Déu, 2. 08950 Esplugues de Llobregat, Barcelona, Spain; Center for Biomedical Research on Rare Diseases (CIBERER), ICIII, Madrid, Spain; Instituto de Investigaciones Biomédicas Alberto Sols, Consejo Superior de Investigaciones Científicas - Universidad Autónoma de Madrid, Madrid, Spain; Biochemical Genetics&Rett Unit, Laboratory Department, Hospital Materno-Infantil Sant Joan de Déu, Barcelona, Spain

**Keywords:** Hypotonic infant, Thyroid hormones, MCT8, Allan-Herndon-Dudley syndrome

## Abstract

**Background:**

Thyroid hormone is crucial in the development of different organs, particularly the brain. MCT8 is a specific transporter of triiodothyronine (T_3_) hormone and MCT8 gene mutations cause a rare X-linked disorder named MCT8 deficiency, also known as Allan-Herndon-Dudley syndrome, characterized by psychomotor retardation and hypotonia. Typically, elevation of T_3_ and delayed myelination in cerebral magnetic resonance imaging are found.

**Case presentation:**

We present a 24-month-old boy, born from non-consanguineous healthy parents, with severe motor and cognitive delay and global hypotonia, being unable to hold head upright or sit without support. Deep tendon reflexes were absent bilaterally at the ankles. T_3_ was elevated and thyroxine slightly decreased, consistent with MCT8 deficiency. Genetic studies confirmed the diagnosis.

**Conclusions:**

Although a rare disease (MCT8 mutations have been reported in about 50 families all around the world), we illustrate the importance of excluding Allan-Herndon-Dudley syndrome in the evaluation of floppy male infants with development delay, without history of perinatal asphyxia. The simple evaluation of thyroid status, including T_3_, T_4_ and TSH can guide the diagnosis, avoiding a number of useless, expensive and invasive investigations and allowing appropriate genetic counseling to the affected families.

## Background

Thyroid hormone (TH) plays a major role in the growth and development of multiple tissues, in particular the brain [1, 2]. The effects of TH are determined by the intracellular concentration of triiodothyronine (T_3_) available to bind to its nuclear receptor [1, 3–5]. Recently, monocarboxylate transporter 8 (MCT8) has been identified as an active and specific TH transporter that plays a critical role in the transport of T_3_ across the blood–brain barrier and in T_3_ uptake into neuronal cells [3, 6, 7]. Different mutations in MCT8 are responsible for a rare X-linked condition, Allan-Herndon-Dudley syndrome (AHDS) that is characterized by global hypotonia that progresses into spasticity with severe psychomotor delay [8]. All affected males present with elevated serum levels of T_3_, low to below normal serum levels of prohormone thyroxine (T_4_) and thyroid stimulating hormone (TSH) in normal range [8, 9].

The authors describe a 24 month-old-boy with severe hypotonia during the first year of life, emphasizing the clinical, laboratory and neuroradiological findings that prompted the genetic study that confirmed the mutation in MCT8 gene.

## Case presentation

We present a 24-month-old male patient, second child of non-consanguineous parents. During pregnancy thyroid hormone levels of the mother showed low free T_4_ level (8.75 pmol/L; normal values 9.39 - 28.31) and normal TSH level (3.17 mUI/L, normal values 0.25 - 5.0). Delivery was uneventful. Gestational age was 38 weeks and the Apgar score in the first and fifth minutes was 9 and 10 respectively. His birth weight and length were normal and the head circumference (38 cm) was above 2SD. Neonatal period was normal.

The mother’s homozygote twin sister has a 6-year-old son with the diagnosis of cerebral palsy with no history of any risk factors (prenatal, gestational or postnatal). The father and paternal grandmother have a sensorineural hearing loss, which occurred at around the age of forty.

From the age of 3 months global hypotonia and poor head control became evident. A rehabilitation program was started without any satisfactory improvement. At the age of 12 months the severe hypotonia persisted, without any improvement in head control. The child was able to grasp objects and bring both hands to the midline, but with significant difficulty. No pyramidal tract involvement was described. At this age, the pediatrician described the child as active and reactive to stimuli, with good visual contact, normal social smile and appropriate interaction between the child and his parents. At that time, cerebral magnetic resonance imaging (MRI) was performed and revealed an enlargement of the subarachnoid spaces, without any description of white matter disorder. Brainstem auditory evoked potential was normal. Assessment of visual evoked potentials showed evidence of a conduction delay in central optical pathways; fundoscopy was normal. No alterations were detected in the basic laboratory and metabolic investigations performed (serum electrolytes, renal and hepatic function, creatine kinase, biotinidasis, urate, lactate and pyruvate, ammonia, urinary organic acid, plasma and urinary ammino acid).

**Figure 1:**
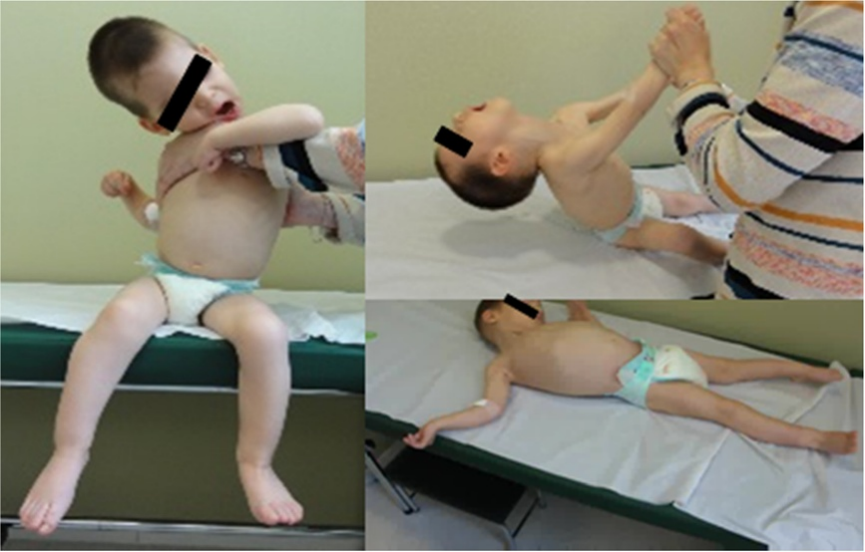
**Clinical features.** The child at 24 months of age showing global hypotonia, hypertonic posture of the limbs, incapacity to sit without support, and to hold the head upright.

By the age of 21 months the patient was referred to our Neuromuscular Pediatric Unit due to severe motor and cognitive delay. At this first visit the patient showed global and severe hypotonia, being unable to hold the head upright and to sit without support (Figure 1). When supine, the patient demonstrated brief episodes of mild dystonic movements and hypertonic posturing of the limbs, triggered by stress conditions or sensitive stimulus. The myotatic reflex was slightly increased in *triceps surae* muscle and Babinski sign was observed. Deep tendon reflexes were absent at the Achilles tendon bilaterally. Eye contact and visual tracking of human face were poor. He was responsive to sounds but was unable to speak.

**Figure 2:**
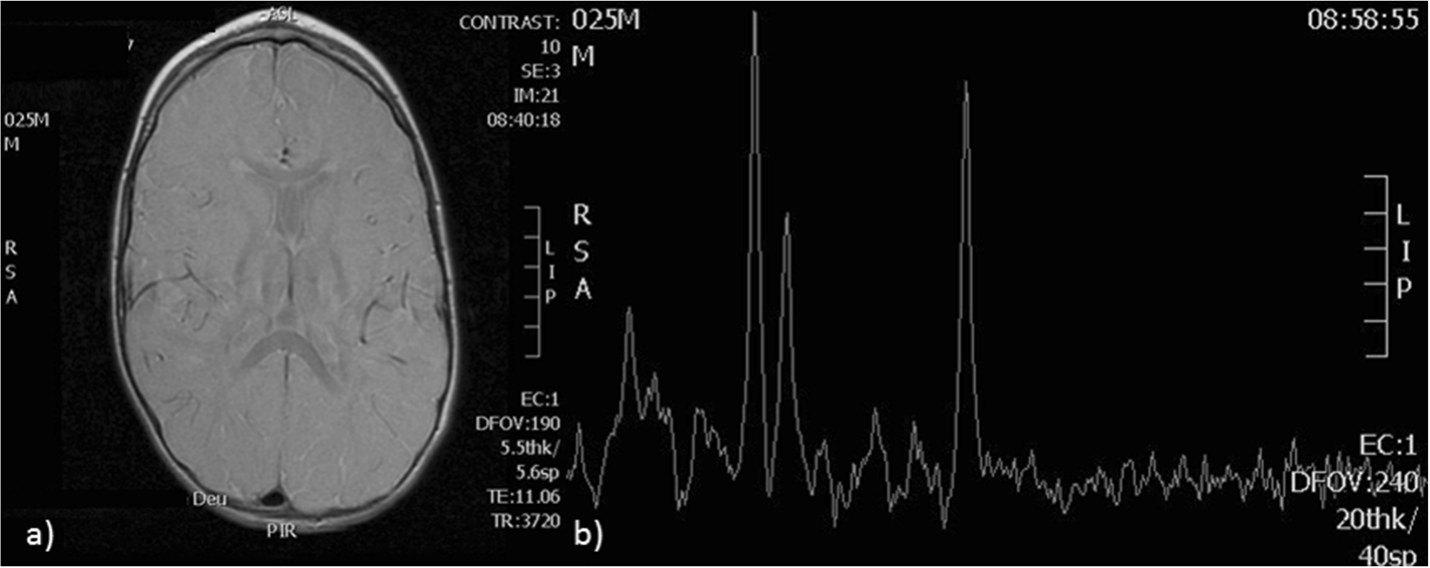
**Cerebral magnetic resonance image.** Axial, T2-fast spin echo sequence **(a)** shows a significative alteration in white matter substance in the *centrum semiovale*, frontoparietal, parieto-occipital and subcortical regions, compatible with a marked delay in myelination; and cerebral magnetic resonance spectrometry **(b)** demonstrates a low N-acetyl aspartic peak.

At the age of 23 months he developed myoclonic jerks characterized by flexion of the neck and upper limbs, lasting no longer than few seconds, ocurring up to twice or three times per day. Initially these epileptic seizures improved with the administration of sodium valproate; however they have reappeared, up to three to four times a day, in spite of the treatment with sodium valproate, levetiracetam and vigabatrin. The latest electroencephalograms revealed polyspike and wave discharges that are widely spread over both hemispheres of the brain with a slow background rhythm. Electromyography and neurography were normal. Electroretinogram was normal. Cerebral MRI was repeated at 24 months of age, which demonstrated a delay of myelination (Figure 2).

**Figure 3:**
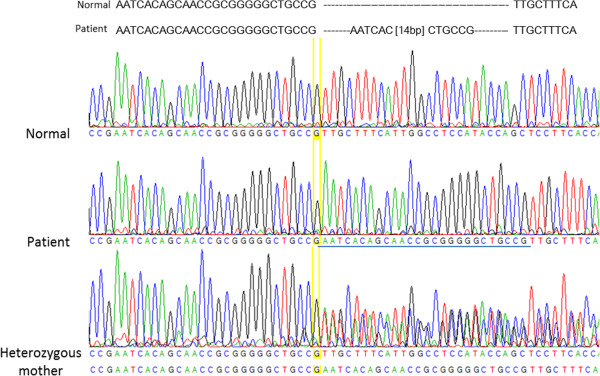
**Electropherograms.** The duplication found in exon 2 of MCT8 in the patient (middle tracing), the sequence of the heterozygous mother (bottom tracing), and the corresponding normal sequence (top tracing).

After repeating some laboratory tests, alterations on thyroid function revealed elevated free T_3_ level (4.84 nmol/L, normal values 0.63-3.90), low free T_4_ level (8.2 pmol/L, normal values 9.1 - 25.0) and normal TSH level (3.54 mUI/L, normal values 0.3 - 4.5). Given this hormonal pattern, the existence of a deficit in T_3_ carrier was considered as a potential diagnosis. To investigate this hypothesis direct sequencing of the SLC16A2 gene was performed, revealing 26 base duplication in exon 2. This gene mutation has not been described previously and causes a Val254Glu substitution followed by a frame shift and a premature stop codon 24 aminoacids later (Exon 2 c.735_760dup p.Val254Glufs*24). His mother was subsequently confirmed to be a carrier for this duplication (Figure 3).

## Discussion

This phenotype of severe X-linked psychomotor delay was first described in 1944 and was eponymously named Allan-Herndon-Dudley syndrome [10]. Afterwards, in 2004, the first mutations in MCT8 gene *(SLC16A2)* were discovered by two distinct investigation groups (Dumitrescu *et al.* and Friesema *et al.*), and neurological findings of AHDS were explained by the resistance of T_3_ on entering the neuronal target cells, that leads to a classical thyroid profile found in all affected patients [8, 9]. Currently, MCT8 mutations have been reported in about 50 families worldwide [4, 11].

Diagnosis of AHDS in our patient is supported by: the presence of an X-linked inheritance; the characteristic thyroid hormonal pattern; the marked delay of myelination of the central nervous system (CNS) found in the MRI and the presence of the pathogenic mutation in the MCT8 gene. AHDS shows a broad and heterogeneous clinical spectrum according to the type of mutation and its impact on the protein synthesis stage. Our patient fits well with the most severe phenotype [12].

One of the main features of AHDS, also known as MCT8 deficiency, is the marked global hypotonia and difficulties maintaining the head up right, recognized as ”limber neck” (Figure 1), evident since the first months of life [13]. In the presence of a floppy infant, evaluation of signs of CNS involvement is essential but not always easy during the first year of life. In this case, normal eye contact and social interaction by the age of 12 months, in addition to the lack of deep tendon ankle reflexes, made peripheral neuromuscular involvement an important hypothesis to exclude. The absence of signs of CNS involvement during the first year of life could have resulted in a muscle biopsy in order to exclude a congenital myopathy or other muscle disorder. However, the presence of spasticity and abnormal movements during the second year of life make it clear that this investigation was not warranted.

During the evaluation of a hypotonic infant, the cerebral MRI is a helpful tool for detecting CNS abnormalities [11]. The difficulty in interpreting a cerebral MRI during the first year of life leads to different descriptions of the white matter abnormalities ranging from delayed myelination to hypomyelination, which can delay the diagnosis [11, 12, 14, 15]. Despite the static or even deteriorating clinical picture for these patients, several studies confirm that the major neuroradiological feature of this disease is the marked delayed myelination [14, 15]. Many of these children were described as a *“Pelizaeus–Merzbacher–Like Disease”* because they had a similar phenotype without the identification of PLP1 mutation. This is a differential diagnosis that needs to be considered and the cerebral MRI is an important tool. In *Pelizaeus–Merzbacher* disease we find a hypomyelination (same pattern of deficient myelination on 2 MRIs at least 6 months apart in a child older than 1 year) while in AHDS, a correct MRI interpretation will show a progression of myelination, even if slow [11, 14]. In our case the relatively low N-acetyl aspartic peak in the cerebral magnetic resonance spectroscopy also supports the hypothesis of delayed myelination (Figure 2) [16].

The neurological manifestations of this disease are quite complex. The axial hypotonia persists throughout adulthood, while the hypotonia of the limbs progresses to spasticity and dystonic posturing [3, 13]. The age of onset of extrapyramidal symptoms is not clear in the literature and it seems essential for us to suspect of AHDS even before these signs appear [13]. Other important features are the inability to sit, stand or walk independently, the severe mental retardation with lack of speech development and rudimentary communicative skills [13, 17]. A quarter of the patients will have seizures that are usually responsive to anticonvulsant therapy [13].

As already mentioned all affected males with MCT8 mutations exhibit a typical thyroid profile which makes it crucial to request these hormones, including T_3_ early in the investigation [3, 8, 9]. Mean serum T4 and free T4 are normal or slightly decreased, TSH is normal or mildly increased and serum T3 and free T3 are markedly elevated [3, 8, 9, 14]. This will help the clinician to avoid unnecessary tests and to pursue a specific genetic analysis of SLC16A2 for a definitive diagnosis. There is another medical condition with the same thyroid hormonal pattern, that can be found in a hypotonic infant and that is due to a mutation in the thyroid hormone receptor α gene (TRα1). This condition has a similar presentation in the first months of life; however older infants and children present with a phenotype very different from AHDS, developing the classic features of hypothyroidism (growth retardation, skeletal dysplasia, reduced muscle tone, constipation) and only a mild cognitive impairment [18].

Some AHDS patients have been treated with TH supplementation, propylthiouracil or diiodothyropropionic acid; the last two result in better thyroid function tests, but without any improvement of motor or cognitive skills [19–21]. Up to this moment, we can only offer symptomatic treatment to these patients, such as rehabilitation therapies, antispasticity, antidystonic and anticonvulsant medication, nutritional and orthopedic management [13, 20, 22]. Genetic counseling should be offered to the family as soon as the diagnosis is recognized as is the case with all other X-linked recessive conditions: if the mother has SLC16A2 mutation, boys will have a 50% risk of being affected, whereas girls will have a 50% chance of being a carrier of the mutation [13, 22]. The genetic test was suggested to be undertaken by the patient’s cousin but unfortunately his parents have systematically refused.

## Conclusion

Through this clinical report we demonstrate the importance of excluding AHDS in the initial laboratory evaluation of floppy male infant, without history of perinatal asphyxia, by the simple exploration of the thyroid hormone status, including T_3_. In spite of the absence of an effective treatment for this disease, an early diagnosis can avoid a number of useless, expensive and invasive investigations and permit correct genetic counseling to the affected families.

## Consent

Written informed consent was obtained from the parents of the patient for publication of this Case report and any accompanying images. A copy of the written consent is available for review by the Editor of this journal

## Abbreviations

TH: Thyroid hormone
T_3_: Triiodothyronine hormone
MCT8: Monocarboxylate transporter 8
AHDS: Allan-Herndon-Dudley syndrome
T_4_: Thyroxine
TSH: Thyroid stimulating hormone
MRI: Magnetic resonance imaging
CNS: Central nervous system
TRα1: Thyroid hormone receptor α gene.
